# Disaster response and older adult cancer care in super-aged societies: insights from the 2024 Noto Peninsula Earthquake in Oku-Noto, Japan

**DOI:** 10.3389/fpubh.2024.1427987

**Published:** 2024-10-24

**Authors:** Ayu Yasui, Seiichi Kinoshita, Michioki Endo, Yudai Kaneda, Ryo Ikeguchi, Chika Yamamoto, Toshiki Abe, Tianchen Zhao, Toyoaki Sawano, Kenji Gonda, Masaharu Tsubokura, Hiroyuki Beniya, Hiroaki Shimmura, Akihiko Ozaki

**Affiliations:** ^1^Breast and Thyroid Center, Jyoban Hospital of Tokiwa Foundation, Iwaki, Japan; ^2^Department of Surgery, Wajima Municipal Hospital, Wajima, Japan; ^3^Department of Surgery, Hyogo Prefectural Awaji Medical Center, Sumoto, Japan; ^4^School of Medicine, Hokkaido University, Sapporo, Japan; ^5^Katsuyama Orange Clinic, Katsuyama, Japan; ^6^Department of Radiation Health Management, Fukushima Medical University School of Medicine, Fukushima, Japan; ^7^Department of Surgery, Jyoban Hospital of Tokiwa Foundation, Iwaki City, Fukushima, Japan; ^8^Orange Home Care Clinic, Fukui, Japan; ^9^Department of Urology, Jyoban Hospital of Tokiwa Foundation, Iwaki, Japan

**Keywords:** older adult cancer patients, super-aged society, disaster medicine, disaster preparedness, oncology care

Natural disasters can severely disrupt cancer care delivery systems, compromising the continuity and quality of oncological services (1). This issue has been a subject of growing concern since Hurricane Katrina in 2005 (2), with the 2011 Great East Japan Earthquake further intensifying research and discussions (3–6). Recent events, including the 2015 Nepal Earthquake (7), 2017 Hurricanes Irma and Maria in Puerto Rico (8), and the 2023 Morocco earthquake (9), have highlighted the critical need for effective cancer management strategies during disasters, particularly in low- and middle-income countries (LMICs). These incidents demonstrate that the impact of disasters on cancer care varies significantly based on their nature, severity, and the resources available in affected regions.

In disaster preparedness and response, protecting vulnerable populations, especially the older adults, is crucial due to their heightened susceptibility to adverse health outcomes (10). This concern is particularly relevant in high-income countries like Japan, where 29.1% of the population is 65 or older (11), and in low- and middle-income countries (LMICs), projected to house 80% of the world's older adults by 2030 (12). While the impact of disasters on older cancer patients has gained attention in academic literature (13), there remains insufficient discussion on managing cancer care in disaster-stricken super-aged societies (where over 21% are 65 or older). This distinction is significant, as super-aged communities may be inherently more vulnerable and struggle to respond to disasters without external support, presenting unique challenges in maintaining cancer care continuity during crises.

Here, we would like to present the case of the Oku-Noto region, as it exemplifies unique challenges for disaster response and cancer care continuity in a super-aged society. This area's older adults reaches an unprecedented 48.9% (14), far exceeding Japan's national average. On January 1, 2024, the Noto Peninsula Earthquake caused significant damage to Oku-Noto in Ishikawa, Japan, resulting in 241 deaths as of February 16, 2024 (15). This event provides a critical case study for examining disaster response in cancer care in super-aged societies.

In Oku-Noto, as was the previous cases (5, 8), the acute phase of the earthquake was the most likely time for treatment interruptions or delays for cancer. In the Noto earthquake's affected areas, many older adults were forced to evacuate outside the region early on due to the subsequent destruction of buildings and infrastructure. Preliminary observations indicate that medical institutions effectively collaborated to maintain cancer care continuity during the disaster's initial phase. Notably, Wajima Municipal Hospital, the primary healthcare facility in Wajima City—which reported 102 disaster-related fatalities—coordinated the referral of numerous cancer patients to external medical facilities. While the exact number of referred patients remains undetermined, this proactive approach ensured uninterrupted care despite challenging circumstances. Such seamless coordination of patient referrals amid a major disaster represents a significant accomplishment in maintaining critical healthcare services, addressing a challenge highlighted during the 2011 Great East Japan Earthquake (5). The second author of this manuscript, professionally engaged at the hospital, provides firsthand insight into these efforts.

Conversely, the region faces pressing challenges in providing medium to long-term care for cancer patients. As nearly seven months have elapsed since the earthquake, and with ongoing infrastructure restoration, older-adult evacuees are gradually returning to the affected areas. Of particular concern are the psychological impacts of the earthquake on cancer patients, as highlighted in a recent scoping review (13). Moreover, considering that the potential physical effects of the earthquake may be more pronounced among the older adults (16), older cancer patients might require more extensive support upon their return.

However, as seen after the Great East Japan Earthquake (17), Oku-Noto is experiencing a significant exodus of medical personnel, particularly nurses. The Noto Peninsula Earthquake has severely impacted healthcare staffing in the region. Reports indicate that by the end of the fiscal year in March, Wajima Municipal Hospital lost approximately 25% of its nursing staff—about 30 out of 120 nurses (18). This trend is not isolated; across the Noto Peninsula, a total of 60 nurses departed from their positions at the four public hospitals in the region (19). Consequently, the total number of available beds in these four public hospitals dramatically decreased from 538 pre-earthquake to just 240 by the end of June—a reduction of more than 55% (20). This substantial decline in both staffing and bed capacity has undoubtedly compromised the region's ability to provide comprehensive medical care, including critical oncological services.

Following the restoration of critical infrastructure by mid-March, Wajima Municipal Hospital has begun incrementally recovering its oncological services, with plans to resume surgical operations as of March 21, 2024, and the reopening of outpatient chemotherapy in April. However, despite these improvements, the area's substantial older adults continues to face challenges reminiscent of those observed after the 2011 Great East Japan Earthquake (5). Limited internet access restricts the older adult's ability to obtain crucial health information, while transportation barriers impede their access to medical treatment both locally and beyond.

In response to the Noto earthquake, the Japanese Nursing Association (JNA) dispatched 2,982 nurses to affected areas from January 6 to February 29 (21). The JNA coordinated with local authorities to meet on-ground needs and recruited nurses for longer-term assignments (1 month to 2 years) in Oku-Noto hospitals, with 13 deployed by July's end (20). This sustainable nurse deployment system aims to address immediate staffing shortages and build long-term healthcare resilience in the region. However, integrating temporary staff and maintaining continuity of care, especially for chronic conditions like cancer, remains a critical consideration.

In this respect, an integrated home healthcare approach offers a viable solution for supporting older cancer patients in disaster-affected areas over the medium to long term. This model requires fewer hospital resources and medical personnel, as healthcare providers travel to patients' homes. While demanding a deeper understanding of local contexts, home-based care alleviates hospital strain, offers personalized treatment in familiar settings, and potentially improves outcomes for older cancer patients. This approach is particularly valuable in areas where traditional healthcare infrastructure is compromised by disasters, providing a sustainable model for older adult cancer care.

Our observations from the Oku-Noto region offer insights that extend far beyond its geographical boundaries. This area serves as a microcosm of Japan's demographic future, potentially mirroring the nation's population structure in ~40 years. Moreover, this scenario is likely to be replicated in other countries as global populations age. In Japan, priorities include: ensuring uninterrupted treatment in isolated regions, developing flexible care networks, addressing medical staff exodus, implementing home healthcare for less mobile older adults, and creating sustainable staffing models through long-term nurse dispatch programs. These measures aim to build a resilient cancer care system capable of withstanding and adapting to disaster situations in an aging society.

Other countries can also adapt Oku-Noto's lessons based on their resources: high-resource nations might prioritize advanced transfer systems and telemedicine, while lower-resource countries focus on basic care continuity and community support. Key strategies include tailoring preparedness to local demographics and resources, and training local healthcare workers in basic oncology care where specialist deployment is challenging. The Noto experience ultimately calls for international collaboration in developing adaptable best practices for maintaining cancer care during disasters in aging populations worldwide.
